# Mapping Nairobi's dairy food system: An essential analysis for policy, industry and research

**DOI:** 10.1016/j.agsy.2018.08.007

**Published:** 2018-11

**Authors:** Stella Kiambi, Pablo Alarcon, Jonathan Rushton, Maurice K. Murungi, Patrick Muinde, James Akoko, Gabriel Aboge, Stephen Gikonyo, Kelvin Momanyi, Erastus K. Kang'ethe, Eric M. Fèvre

**Affiliations:** a Department of Public Health, pharmacology & Toxicology, University of Nairobi, Nairobi, Kenya; b International Livestock Research Institute, Nairobi, Kenya; c State Department of Veterinary Services, Kenya,; d Veterinary Epidemiology, Economics and Public Health group, Royal Veterinary College, Hatfield, United Kingdom; e Institute for Infection and Global Health, University of Liverpool, Liverpool, United Kingdom; f State Department of Livestock Production, Kenya

**Keywords:** Dairy, Urban, Kenya, Value chain analysis, Mapping

## Abstract

Demand for dairy products in sub-Saharan Africa, is expected to triple by 2050, while limited increase in supply is predicted. This poses significant food security risk to low income households. Understanding how the dairy food system operates is essential to identify mitigation measures to food insecurity impact. This study aims to determine the structure and functionality of Nairobi's dairy system using a value chain mapping approach.

Primary data were gathered through focus group discussions and key informant interviews with dairy value chain stakeholders in Nairobi to obtain qualitative information on people and products in the chains while describing their interactions and flows. Qualitative thematic analysis combined with flowcharts created by participants enabled identification of key food system segments and the development of chain profiles (or flow-diagrams) which together form Nairobi's dairy system.

Seven chain profiles forming Nairobi's dairy value chain were identified. These were found to be dominated by small-scale individuals who operate largely independently. Our profiles for the urban and peri-urban farming systems were structurally similar in their downstream networks, obtaining inputs from similar sources. Upstream, the urban systems were shorter, supplying mostly to immediate neighbours or based on own consumption, while the peri urban systems supplied to a wider network and showed some affiliations to producers' associations. Two distinct profiles characterize the milk flow from traders belonging either to a Dairy Traders Association (DTA) or those not belonging to this association (non-DTA). DTA traders sell mainly to fixed retailers and non-DTA traders to mobile retailers (hawkers or roadside vendors). Profiles associated with medium and large cooperatives were driven by networks of collection centres, but with medium-sized cooperatives selling half of their production to large processing companies, and large cooperatives only to fixed retailers. Large processing companies' profiles indicated distribution of high volumes and value addition processing. They reported strategic milk collection arrangements with suppliers on long, medium - or short - term contracts and with well-established product distribution channels.

We have identified numerous inter-linkages across dairy chain profiles in Nairobi's complex system, demonstrating significant interdependency among the stakeholders. Therefore, enhancing the system's efficiency requires a holistic, system-wide approach and any policy interventions should consider every segment of the value chain. This study provides a methodological approach for organizations and policy makers to understand and address structural and functional vulnerabilities within food systems more broadly. The insights from this study are relevant to other rapidly growing cities in the region.

## Highlights

• The structure of Nairobi’s complex dairy value chain is provided.
• Inter-dependency and inter-linkages of the stakeholders involved in the dairy chain is described
• To enhance the system’s efficiency, interventions require a holistic approach targeting every segment of the value chain

## 1 Introduction

Global demand for dairy products has gained prominence over the past few decades due to population growth and increase in per capita income in developing countries (Herrero et al., 2014), coupled with alteration of the global supply that has been influenced by significant changes in husbandry, genetics and nutrition linked to new processing and marketing systems. By 2050, it is estimated that in sub-Saharan Africa milk demand will triple with the greatest increases in East Africa (Herrero et al., 2014). However, milk supply across the region is not predicted to match the estimated demand. An in-depth consideration of milk value chains to identify strengths and weaknesses of the existing systems to estimate how they will respond to the shortfall in supply is critical.

In 2012, Kenya, the country with the highest per capita milk consumption in Africa (SDP, 2004), produced about 4.8 billion litres of milk (FAOstat, 2012); 75% was obtained from cows, 18.8% camels, 5.4% goats and 0.7% from sheep. The dairy sector is one of the largest agricultural segments of the country contributing about 4% of the national Gross Domestic Product (GDP) and 14% of the agricultural GDP (KDB, 2014). The industry which was initially monopolized by the government through the Kenya Cooperative Creameries (KCC) has rapidly evolved following its liberalization and decontrol of prices in the 1990s (Leksmono et al., 2006) resulting in an explosion of informal dairy markets while generating many opportunities for private processors (Muriuki et al., 2003). Growing at an annual rate of about 5 to 7%, the sector is a source of livelihood to roughly 1.8 million small-scale producers who account for over 80% of the country's milk producers (KDB, 2014). The marketing channels are mainly driven by the informal sector which is responsible for over 70% of all marketed milk (FAO, 2011a). This translates to over 40,000 employment opportunities which are approximately 70% of personnel working in the dairy industry in Kenya (FAO, 2011a).

Government annual reports on milk production indicate that milk production within Nairobi accounted for about 37 million litres per year (unpublished government milk production data, 2012). Conversely, milk intake is estimated to be highest in the urban centres at 125 l per capita (SDP, 2004). This implies that Nairobi, with a population of about 3.1 million people (KBS, 2010) consumed more than 388 million litres of milk in 2009 or approximately 10% of the country's production. Thus, over 90% of milk consumed in Nairobi is supplied through value chains linked to production outside the city. Understanding the structure and functionality of such milk chains is essential.

A few studies have attempted to describe the structure of the country's dairy value chain (Baltenweck et al., 1998; Staal et al., 2001; TechnoServe, 2008; Rademaker et al., 2016). However, the methodologies used have been on general flows rather than a comprehensive description of each of the specific segments of the dairy value chain, which is critical in understanding the overall dairy system.

The current study utilizes the ‘Mapping’ component which is one of the four critical steps in conducting a value chain analysis (VCA) (Kaplinsky and Morris, 2000; FAO, 2011b). Mapping involves a systematic analysis of the people involved and products flow along the value chain taking into consideration input supply, production, processing, distribution and marketing activities of a specific product or service (Kaplinsky and Morris, 2000). It provides a visual depiction of the basic structure and a framework to guide systematic chain analysis and other important areas such as food safety and pathogen flows (Alarcon et al., 2017). The aim of this study was to identify and assess the structure and functionality of the Nairobi's cattle dairy value chain.

## 2 Materials and methods

This cross-sectional study was implemented in Nairobi County between January 2014 and January 2015. The research questions investigated in this study were: 1) Who are the people (and organizations) involved in the Nairobi's dairy value chain? 2) What is the structure of the milk production and milk flow into the city? 3) What is the overall structure of the Nairobi's dairy value chain? and 4) What are the factors that define the interaction of different stakeholders? The mapping methodology used in this study is based on (Alarcon et al., 2017).

### 2.1 Study area

**Fig. 1:**
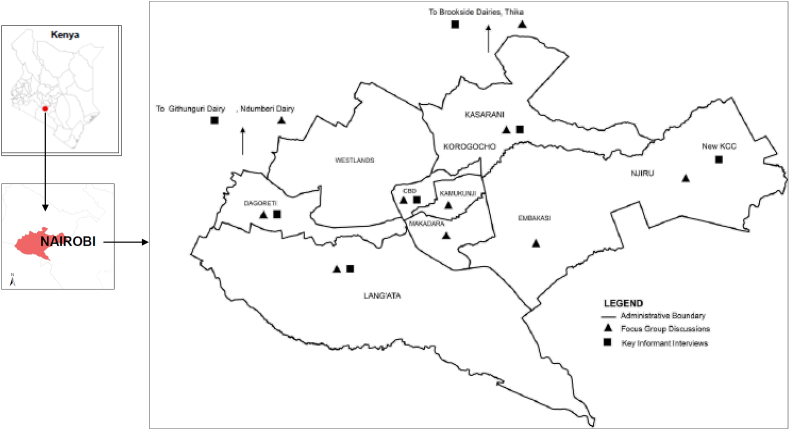
Map of Nairobi County showing administrative boundaries and study sites.

Nairobi County, the capital city of Kenya, is the second largest city by population in Africa's Great Lakes Region after Dar-es-Salam and is the 13th most populated city in Africa (CIA, 2014). With a population of >3.1 million multi-ethnic residents, Nairobi hosts approximately 8.1% of the country's total population (KBS, 2010). With the projected annual growth rate of 4% (Aubry et al., 2010), Nairobi will be home to >5.7 million people by 2030 and approximately 8.2 million people by 2050. The County is divided into nine sub-counties (Fig. 1). Dairy farming is practised in all the sub-counties (unpublished government milk production data, 2012). Kasarani and Lang'ata sub-counties produce the highest quantities with an average annual production of >12 million litres while Embakasi, Makadara and Kamukunji produce less than one million litres per year.

### 2.2 Selection of participants

A stakeholder analysis was done through a detailed desktop review to identify the main organizations and people involved in the dairy value chain and to determine the process of data collection.

#### 2.2.1 Key informant interviews

Key informant interviews (KIIs) with relevant senior staff at the Directorate of Veterinary Services (DVS), Directorate of Livestock Production (DLP) and the Kenya Dairy Board (KDB) were done to further identify and validate the developed list of key people and organizations, and to generate an initial flow diagram of the dairy system in Nairobi. Broad consultations with other researchers who were or had previously worked on dairy value chain studies were done to improve on this stakeholder analysis. These included; United States Agency for International Development dairy value chain competitiveness program; International Livestock Research Institute and the Kenya Agricultural and Livestock Research Organization. At the end of each interview, the key informants were requested to suggest another person(s) who could be asked the questions that they could not handle adequately. They also suggested other companies or sectors that were viewed to play an important role in the system (snowballing interview process).

#### 2.2.2 Focus group discussions

Selection of participants in each group was based on their specific type of enterprise and interviews conducted independently to each group. Whenever possible during the focus group discussions (FGDs), representation for both males and females was ensured to account for gender differences.

### 2.3 Data collection

Twenty FGDs with 105 people and 23 key informant interviews with 35 people were conducted (Annex 1). Secondary data from the Department of Livestock Production was analysed to understand the production systems within the city. Primary data were obtained through FGDs, key informant interviews (KIIs) and researchers' observations. Prior to engagement of the participants, written consent was sought and obtained and agreement on the preferred language (s) for discussions. A minimum of two research assistants recorded the discussions in notebooks and a backup of the audio and video recordings.

In each FGD, local person who understood the local language (s) was identified to clarify words or statements unclear to the group. Participants could brainstorm on each question until there was consensus on the issue under discussion. The facilitator-utilized flipcharts to draw the flow of people, livestock and products as the participants described them. Where possible, the participants were asked to agree on proportions of people, livestock and products within a specific chain; otherwise, they were asked to agree on the main pattern. The facilitator ensured frequent prompts to further explore and clarify the activities, people and products flow. An interviewer administered open-ended questionnaire was used to ask participants in each FGD to describe the enterprise operations, actors, differences in sourcing characteristics, type of livestock and products traded/kept, modalities of engagement and interaction, source of farm inputs and waste management practices.

Similar questions were asked to each of the key informants but additionally describing their interaction with the government and other stakeholders, role in influencing the chain, products description including their flows. Secondary data supplemented data obtained on dairy cow keeping and milk production in the city.

### 2.4 Data entry and analysis

The voice and video recordings were carefully listened to and all the information was transcribed into pre-formatted templates; which were word documents systematically organized to enter qualitative data in distinct sections based on the emerging themes. Data entry was complemented with data collected in notebooks and on the flip charts created with participants during the FGDs and KIIs.

Thematic qualitative analysis was performed to identify the emerging themes that describe patterns of operations, interactions of people and flow of commodities, inputs or the end disposal of waste. Using these emerging themes and the flowcharts obtained in each FGD and KIIs, advance flowchart (maps) were created to represent the structure of the different chains existing in the dairy value chain. These maps or flow-diagrams are referred here as ‘Chain profiles’. Each chain profile describes in detail a specific segment of the dairy food system. For the purposes of clarity, some of the information such as feeding, watering of livestock, animal health, breeding services, regulation and licensing was omitted from the flowcharts but then explained in the narrative.

Data validation was achieved by ensuring proper representation of the participants following stakeholder analysis. Information gathered through FGDs was triangulated during KIIs. When discrepancies were detected, additional consultations were done with other experts working or conducting research in the dairy value chain.

## 3 Results

**Fig. 2:**
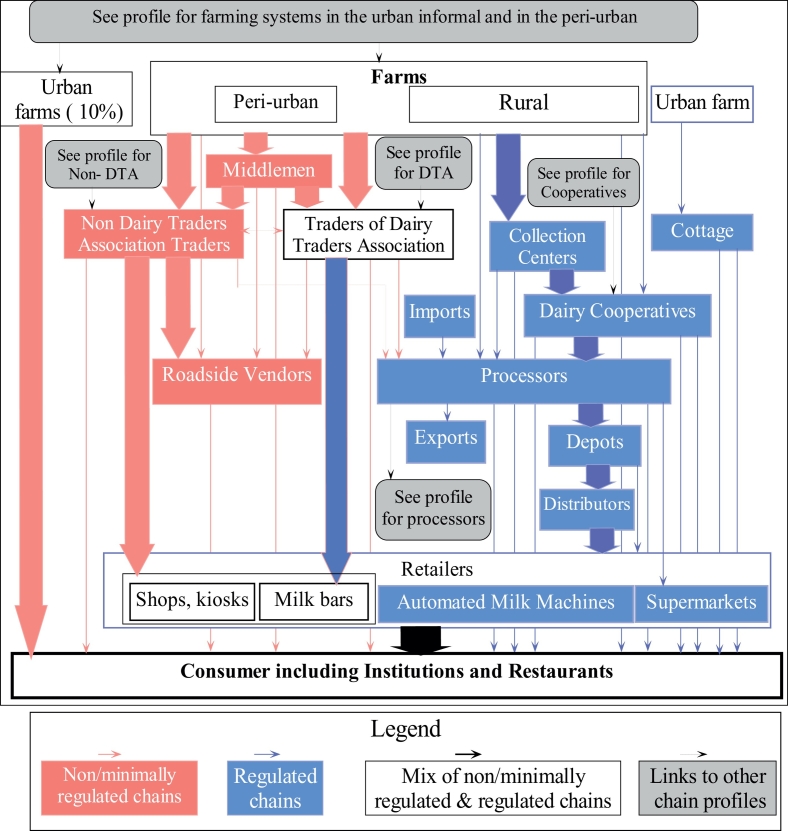
Flow diagram indicating the overall structure of the dairy food system operating in Nairobi. The figure identifies the major chain segments (or chain profiles) composing the dairy system, and which are then provide in full detail in the other figures. This figure differentiates between the non or minimally regulated chains (informal – in red) and the regulated chains (formal – in blue). (For interpretation of the references to colour in this figure legend, the reader is referred to the web version of this article.)

**Fig. 3:**
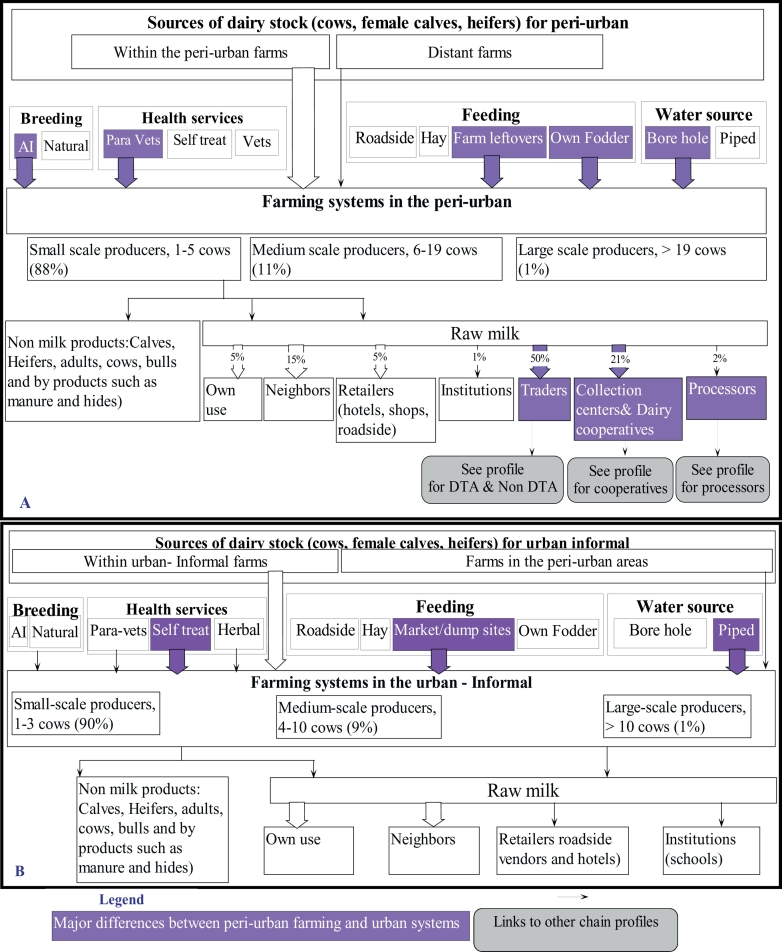
Chain profiles for (3A) peri-urban and (3B) urban farming systems. Footnote: Fig. 3A shows that majority of farms in the peri-urban areas have between 1 and 5 cows; rely on artificial insemination (AI) for breeding, para-veterinarians for animal health services; and their feeding is mainly from farm leftovers and own grown fodder, while water for livestock is mainly drawn from boreholes. Milk selling from these farms is mainly through traders, milking collection centers, diary cooperatives or directly to the large processing companies. Fig. 3B shows that 90% of dairy farming systems in the urban informal areas have between 1 and 3 cows; AI and natural breeding are almost equally utilized; their sick animals are treated by owners without consultation of veterinarians or para-veterinarians; and livestock feeds are mainly obtained from the dumping sites, while water is obtained from taps connected by the city council. Milk selling from these farms is mainly for own consumption or is sold to neighbours.

**Fig. 4:**
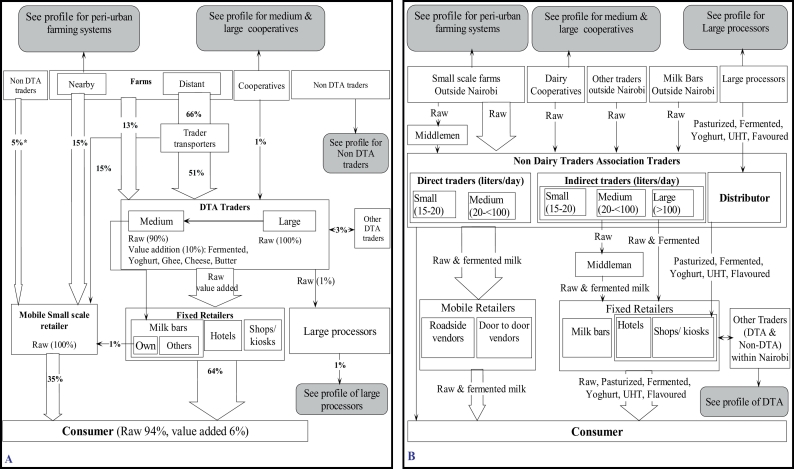
Chain profile for A) traders that are part of the Dairy Traders Association (DTA Traders) and (B) for traders not belonging to the Dairy Traders Association (Non-DTA trader). In the left figure, the percentages indicate the quantity of milk traded by DTA traders and mobile small-scale retailers.Government officials approximated to 30,000 the number of milk traders operating in the country, which then they broadly were categorized into DTA and non-DTA traders. DTA traders form about 20% of the traders in the country and comprise 30% of farmers-traders, 60% of traders-only and 10% trader-transporters. To register with DTA, traders were required to pay registration fee and an annual retention fee. Additionally, though not completely mandatory, traders were required to undergo training by specific KDB accredited business development service providers on milk handling, hygiene, bookkeeping, business ethics and value addition ($20 per course). Once trained, the traders obtained an identification card bearing the DTA and Kenya Dairy Board logos as an identification of legalized traders and hence shielding them from arrests by KDB for illegal milk trading. According to the officials, DTA traders were perceived to provide better quality milk than non-DTA traders. However, the officials estimated that only 45% of their members had gone through the training because traders did not find much benefit in paying for the training since it was still possible to run milk business without it.

**Fig. 5:**
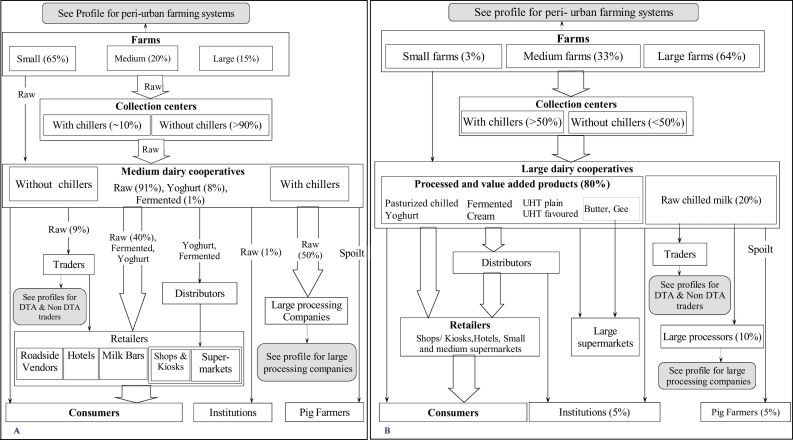
Chain profile A) shows that medium size cooperative gets most of their milk from small scale peri-urban farms and sell it as raw to retailers and large processing companies. On the other hand, B) large size cooperative get their milk form medium and large peri-urban farms and sell most of it to retailer either directly or through distributors.

**Fig. 6:**
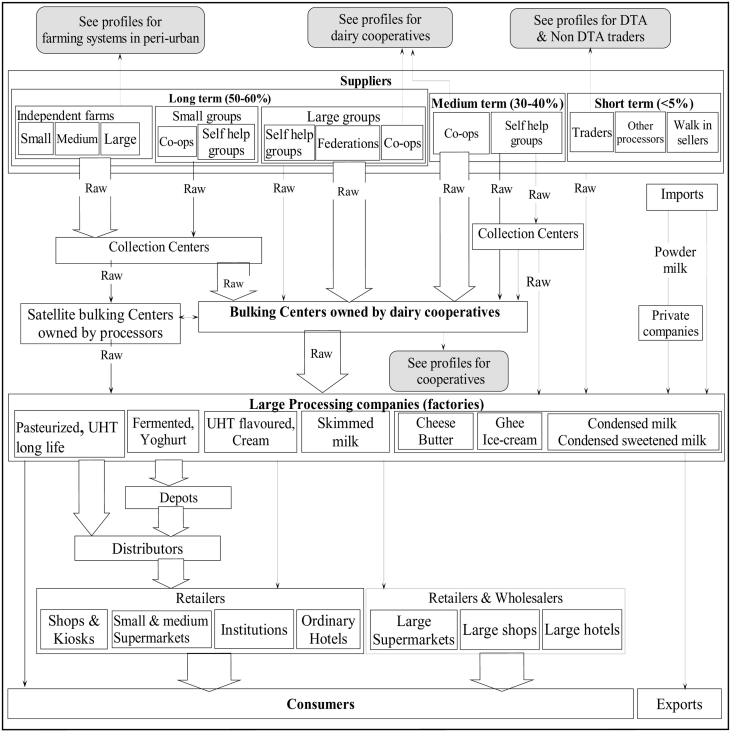
chain profile for large processing companies shows that milk is mainly sourced directly from farmers through collection centres and bulking centres (dairy cooperatives). All milk products emanating from such systems is processed into value added products and sold to retailers through distributors.

Seven chain profiles (or system segments) were identified forming the overall Nairobi's dairy value chain (Fig. 2). These include: farming systems in urban informal and peri-urban areas (Fig. 3 A, 3B); chain profiles for traders affiliated to Dairy Traders Association (DTA) (Fig. 4A) and non DTA (Fig. 4B); medium and large dairy cooperatives (fig. 5A & 5B); and the chain profile for large processing companies (Fig. 6). Each of the chain profiles links to other chain profiles thus forming the overall complex dairy value chain.

Interviews with the government officers revealed that the dairy value chain is comprised of formal and informal chains. Formal chains described as those operated by dairy enterprises that were fully or partially effectively regulated through inspection and licensing. Such chains included dairy cooperatives, milk processing companies, some milk bars, some of the traders within DTA, some shops and one cottage (a type of node where milk is produced, processed, branded and packaged at the farm, mainly for high-class users and large hotels). Informal chains were described as those operated by dairy enterprises that evaded regulation and engaged in minimal value addition activities. Such chains included the roadside vendors, some of the DTA traders, non-DTA traders, some milk bars, some shops and kiosks. Successful operations of the informal enterprises, and particularly with traders, activities were reported to be performed during the night or very early in the morning, away from the official working hours of KDB inspectors.

Detail assessment of each of the segments demonstrates numerous linkages between the formal and informal chains through buying and selling activities resulting to a thoroughly interlinked system. The linkages are described in more detail later in this article.

### 3.1 Chain profiles for the farming systems in the urban informal settlements and at the peri-urban areas of Nairobi

The chain profiles for the two farming systems are shown in Fig. 3.

#### 3.1.1 Dairy herd and livestock keepers

FGDs with livestock production officers estimated approximately 38,000 dairy cow keepers in six of the seven sub-counties of Nairobi County, predominantly in a smallholder setup. Small-scale production in the urban informal was defined as those keeping 1–3 cows (90% of the dairy cow keepers), while in the peri-urban they kept 1–5 cows (88%). Medium-scale farmers kept 4–10 cows (8%) in the urban informal and 6–19 cows (11%) in the peri-urban while large-scale farmers kept >10 cows (1%) in the urban informal and >19 cows in the peri-urban (1%). The large scale and some of the medium-scale farmers were either schools, company farms or individuals who focused on dairy as a commercial enterprise. The small-scale and some of the medium-scale farmers kept dairy cows not only for subsistence but also as a source of prestige and financial security.

Farmers in the urban informal settlements and majority of peri-urban farmers operated independently. However, some farmers in the peri-urban areas were affiliated to self-help groups, livestock producer associations and the dairy cooperative societies. Affiliation to groups was done with the aim of increasing milk production, increasing their bargaining power, creating new avenues for selling milk, as well as to benefit from credit facilities on animal feeds, household items, school fees and organized animal health extension and breeding services.

#### 3.1.2 Sourcing of the dairy stock

Long calving intervals, sometimes lasting more than three years, characterised dairy cows in both urban informal and peri-urban areas. Farmers retained a female calf in the herd unless there was lack of space or an urgent need to sell. Replacement stock were directly bought from neighbourhoods and rarely through brokers.

In the absence of neighbourhood sources or of the need to upgrade to increase milk yields, the urban informal dairy keepers sourced their dairy stock from the peri-urban farmers, primarily through brokers. Heifers were rarely purchased from the livestock Agricultural Showgrounds during livestock exhibitions or from rural areas. Although perceived to be more productive, these sources were alleged to be expensive and posed transportation challenges.

Rumours in the villages were described as the main source of information for identifying buyers for animals. Farmers from both systems preferred to purchase adult dairy cows in their sixth to seventh month of pregnancy or lactating animals as milk is their main source income. Some farmers reported to book a calf from a neighbour's in-calf cow, but with the condition to purchase it only if the calf was a female. Transportation of replacements was done by trekking when these were sourced from neighbourhoods or by vehicles when sourced from long distances.

#### 3.1.3 Housing, feeding, breeding and animal health services

Detailed information is on annex 1. Both the urban informal and peri-urban farmers described their farming systems as zero grazing. Peri-urban dairy farmers moslty utilized own grown fodder (*Pennisetum purpureum*) and garden leftovers (maize stocks and banana stems) as livestock feed while the urban informal farmers reported to mainly use leftovers from markets and garbage from dump sites. Water provision for the dairy stock within urban informal settlements was mainly from piped water supplied by the city council, although sometimes wastewater derived from washing clothes or utensils was used. Peri-urban farmers used water mainly from boreholes and sometimes from piped water, shallow wells and stored rainwater.

Artificial insemination (AI) was the preferred method of breeding; but it was common practice in both farming systems for farmers to opt for natural breeding following frustrations (repeat breeding) from AI. Animal health services were provided principally by animal health assistants in the peri-urban areas and by self-treatment in the urban informal areas. It was mentioned that in both systems obtaining treatment from a veterinarian (a degree holder) was difficult mainly because farmers did not know them, or they were seen as expensive. Medicines were mainly purchased from Agrovets (retailing shops for agricultural chemicals and veterinary medicines) or use of herbal remedies.

#### 3.1.4 Marketing of milk from the urban informal and peri-urban farming systems

Fresh milk was identified as the most important commodity derived from these farming systems. Other products included calves, heifers, bulls, fermented milk and to a lesser extent yoghurt. In both systems, milk was mainly for subsistence use and a source of quick cash through farm gate sales. Farmers explained that the type of agreement between themselves and buyers was purely based on trust and verbal agreement on quantities of milk sold, time for collection, prices and modalities of payment (whether cash, payments in advance or paying after consumption of the milk). However, written agreements prevailed for milk deliveries for peri-urban farmers affiliated to dairy cooperatives.

Over 99% of the milk produced in the informal urban systems was sold raw at farm gate with <1% delivered to local schools and restaurants. Farmers in the peri-urban reported that farm gate sales accounted for >50% of their milk to traders, 15% to neighbours and 5% to retailers. Approximately 30% of their milk was sold through milk collection centres or dairy cooperatives (21%) or directly to the large dairy processing companies (2%). Collection centres were described as specific points or simple sheds located by the roadsides or points under specific trees with or without any structure where farmers deliver milk at a specific time for collection by the dairy cooperative or large processing companies. They were reported to be organized by farmers in those particular localities.

Milk from farms was mainly packaged into recycled plastic containers (soda, water, or beer bottles for smaller volumes and five-litre or twenty-litre plastic containers for larger milk volumes). Those delivering to the collection centres, dairy cooperatives and processing companies were required to use legally recommended aluminium cans. When distances were within the neighbourhoods, milk transportation was mainly done by foot but longer distances particularly in the peri-urban areas involved use of donkeys, vehicles (private and public), motorcycles or motorbikes.

### 3.2 Chain profiles for traders affiliated to Dairy Traders Association and Traders not affiliated to Dairy Traders Association

The chain profiles for DTA and non-DTA traders are shown in Fig. 4A and B respectively.

The non-DTA traders (characterised by non-recognition by KDB and DTA) represent most of milk traders in the country. Their involvement in milk trading was described as “not planned” and of low initial capital investment.

#### 3.2.1 Milk sourcing by DTA and non-DTA traders

There were no major differences between milk sourcing practices by the DTA and non-DTA traders except that some of the non-DTA traders principally dealt with processed branded products such as pasteurized milk, UHT, yoghurt, fermented milk, cheese, ghee and butter. Sourcing of these products was through an organized system and dependent on supply from processing companies.

Overall, it was established that both types of traders obtained raw milk from the same geographical areas and their milk sourcing practices were similar. Almost all the milk handled by the traders was reported to originate directly from farms (mainly small-scale farmers keeping 1–5 cows), and rarely from other sources. Sourcing from dairy cooperatives or from other traders was principally done only to address any deficit in milk volumes for their specific clients.

Approximately 60% of all milk traded in Nairobi by DTA traders was reported to originate from distant farms while large volumes flowed from peri-urban farms outside Nairobi through non-DTA traders. The rest of the milk was believed to originate from farms located in far rural areas of the country. Payments were reported to be done in advance, on cash, weekly or monthly depending on the agreement between the trader and the farmer (s). When agents were involved, monies were channelled to the farmers through them. In this case, the traders, did not interact with the farmers directly.

The main mode of milk transportation by both types of traders was described to be by foot when moving over short distances but mainly by sticking 20-l plastic containers under passengers' seats in the public vehicles for longer distances. Some of the medium and large-scale traders, however, were reported to use their private vans, bicycles or motorcycles to transport milk.

#### 3.2.2 Milk selling by traders in dairy traders association and non-dairy traders association

Both DTA and non-DTA traders sold their products mostly to private consumers and retailers (milk bars, restaurants, shops and kiosks). Sometimes, but rarely, some of the milk was said to be sold to large processing companies or to other traders. It was estimated that approximately 90% of all the milk was sold raw except for the few non-DTA traders dealing with processed branded products. About 30% of medium scale DTA traders were believed to own milk bars and carried out value addition activities on 10% of their milk to produce yoghurt, fermented milk, cheese and butter

No geographical restrictions were reported on where traders are can sell their milk, except with the legal licensing requirement from KDB which only a few traders complied with. However, it was mentioned that traders had clientele who were somewhat “permanently” engaged with them through verbal agreements.

Mobile retailers were described as those traders or retailers without permanent premises. They sold milk by moving from door to door or sold at certain points along the roadsides. For non-DTA traders, milk flowing through mobile retailers was estimated to be considerably higher compared to the flow of milk through fixed retailers. Medium and the large-scale non-DTA traders reported that once they have delivered milk to their main clients (fixed retailers), they sold their remaining milk as roadside vendors. Some large-scale traders were also reported to transport large quantities of milk from far in their vans to sell to passers-by.

Whether from DTA or non-DTA, spoilt milk was never discarded. This was sold majorly as fermented milk at a small price or was converted to yoghurt by adding flavours and food colours to the fermented milk.

### 3.3 The chain profiles for the medium and the large dairy cooperatives

Chain profile for the dairy cooperatives are shown in Fig. 5A and B, respectively. Dairy cooperatives were described as the organizations formed by several farmers who get together to organize corporate bulking (sometimes cooling), value addition activities, distribution and selling of their milk products. Dairy cooperatives were reported to be in the peri-urban areas with none existing in the urban or in Nairobi informal settlements since almost all milk produced by these farms was reported to be sold through farm gate sales. Key informant interviews with dairy cooperatives managers classified these cooperatives into three types, small, medium and large based on the amount of milk handled per day. They were estimated to handle 10,000, 25,000 and 200,000 l per day respectively.

#### 3.3.1 Sourcing of milk by the dairy cooperatives

Milk was reported to be obtained from within the peri-urban farms (Fig. 5). In some cases, farmers approached the cooperatives for enrolment through formation of milk collection centres. Over 64% of members belonging to the large-scale dairy cooperatives were large-scale farmers keeping >19 dairy cows and producing an average of 15–25 l per day while the medium-scale cooperatives membership constituted of approximately 65% of small-scale farmers (2–5 dairy cows) and 20% medium-scale farmers (6–10 dairy cows).

However, whatever scale of the cooperative, milk was directly sold by the farmers to the cooperative through milk collection centres (Fig. 5). Most (>90%) of the collection centres under the medium scale cooperatives were reported to lack chilling facilities while >50% of those operated by large cooperatives were reported to have chillers.

Farmers reported that milk transportation to collection centres involved a variety of transport modes, including by foot, donkeys, bicycles, motorbikes and private or public vehicles. Transportation from the collection centres to the cooperatives was reported to be organized by the cooperatives, but its cost was transferred to farmers in form of revenue deduction from their monthly sales. Payment of milk deliveries was described to be paid by the cooperative at the end of the month into the farmer's bank account.

#### 3.3.2 Milk selling by the dairy cooperatives

Large cooperatives estimated 80% of all their milk was processed to produce pasteurized, UHT milk, yoghurt, fermented, cheese, butter, ghee, cream and long life flavoured milk. These were sold mainly through distributors to retailers or directly to consumers. The remaining 20% was sold raw, but chilled, to other processors (mainly large processing companies), institutions like schools and rarely to traders.

Medium-scale cooperatives sold approximately 91% of their milk as raw to retailers, traders and private consumers. Fermented milk and yoghurt were sold from approximately 8% and 1% of their milk, respectively.

Rejected milk at the cooperative (or collection centre), was normally taken back by the farmer, who then sells it to private consumers and neighbours at a lower price. In some instances, especially if milk spoils after receiving it from farmers, the cooperative reported to offer or to sell it at lower prices to pig farmers.

### 3.4 Chain profile for the large processing companies

Large processing companies (Fig. 6A and 6B) reported to be receiving approximately 400,000 to 500,000 l per day; the actual names of the companies have been withheld.

#### 3.4.1 Milk sourcing by the large processing companies

Milk procurement by both companies revealed similarities in various aspects, such as the strategic milk collection arrangements throughout the milk-producing areas in the country. Milk sourcing for large processing companies was said to mainly depend on contractual arrangements with suppliers, either on a long, medium or short term. About 50–60% of the milk suppliers were contracted on a long-term basis, with yearly renewable contracts. The long-term suppliers enjoyed a pre-established pay per litre throughout the year (irrespective of any unforeseen negative externalities), yearly bonuses, first priority during glut period, regular extension and AI services, credit facilities and prompt payments at the end of the month. Majority of the long-term suppliers are large individual farmers (>19 cows) and farmers' groups (cooperatives, collection centres, self-help groups) of which about 89% comprised of small-scale farmers (1–5 cows).

Medium and short-term suppliers constituted 30% to 40% of the suppliers. They enjoy some of the benefits availed to the long-term suppliers but considered second and third priority for medium and short-term, respectively. Contracts for medium and short-term suppliers lasted about six and three months, respectively. They constituted of small and medium dairy cooperatives, self-help groups and other processors who were unable to finish their raw milk.

Non-contracted suppliers represented 5% of these companies' milk supply. They sold milk to large processors when they have no other place to sell, and especially during wet seasons when there is milk surplus due to overproduction. Some of the non-contracted suppliers include some individual farmers, milk traders, some self-help groups; walk-in sellers and other milk processors. In the case of unavailability of funds to pay suppliers at the end of the month, this category of suppliers is paid last (sometimes payments are done after two or three months).

Due to high competition of the scarce milk at the farm level, especially during the dry seasons (with traders and other processing companies), the large processing companies have arrangements with specific dairy cooperatives, whose supply accounts for >60% of the milk handled by the processors.

Although no specific people are contracted to deliver milk for specific value-added products, milk from pastoral communities was said to be best for cheese and butter production due to its high butterfat and solids-non-fat characteristics. One company also mentioned to import powder milk, which was mainly reconstituted and sold as liquid pasteurized milk particularly during the dry seasons when milk production was low.

#### 3.4.2 Milk marketing by the large processing companies

Large processing companies carry out value addition in all their milk, as shown in the chain profile (Fig. 6). Milk distribution from the factory is done by the companies directly to depots from where wholesalers (distributors) collect it. However some of them were reported to collect the products directly from the companies. Distributors were described as traders with special arrangements with the large processing companies so that, after making down payments to the company, they can collect the company's products and distribute them to retailers (shops, restaurants and supermarkets). Some of the distributors were said to be stationed within the premises of the large processing company.

## 4 Discussion

The objective of this study was to understand the structure of the dairy value chain in Nairobi utilizing the Mapping component of the VCA (Kaplinsky and Morris, 2000; Hellin and Meijer, 2006). Available literature on the Nairobi or Kenya dairy value chains can be traced back in the 1990s (Baltenweck et al., 1998; Gitau et al., 1994). These studies broadly analysed the production node of the dairy value chain in the context of market access and agro-ecological zones. Other studies that followed broadened the analysis scope to include more chain segments and a broader contextual analysis (Staal et al., 2001; USAID, 2008; IFAD, 2012). Presently, VCA has been adopted for analysis of animal disease risks (FAO, 2011b), policy analysis (Leksmono et al., 2006; Muriuki et al., 2003; Schmitz, 2005; Salasya et al., 2006; Kaitibie et al., 2010) and production (Devendra, 2001; Salami et al., 2010) for strategic decision making. The study presented here shows a detailed analysis of the structure of the different elements of the dairy system supplying Nairobi, which has not been fully described before. The results provide an analytical framework to conduct a full dairy value chain analysis and allow effective investigation of food safety risks and policy interventions at a city or countrywide scale. Upgrade recommendations based on the finding of this study are shown in Table 2.

Our findings indicate that Nairobi's dairy value chain is vast, with profound complexities explained by the tightly or loosely interwoven chains. For example, direct or indirect linkages were shown to exist between small-scale and large-scale enterprises as well as between informal and formal chains. While such networks provide business opportunities for all the stakeholders involved, the distinctive flows and interactions provide opportunities for further interrogation to understand food safety and food security issues. This type of examination would be dependent on a detailed understanding of every specific chain considering that each of the chain profiles and their segments have different food safety risks practices, perceptions and controls. Likewise, the numerous inter-linkages identified throughout the system means that interventions and policies supporting some chains may have an impact on other chains. For example, if any policy intervention targets improvement of milk bacterial quality, assessment of the various nodes of the value chain (production, bulking, processing and marketing) would inform on the areas of vulnerabilities that would require certain types of intervention. If, at production level, the milk is contaminated beyond the maximum total bacterial counts, it means the milk that reaches the processor would require prolonged pasteurization procedures which would impact on the profits that the processor may not be ready to incur, unless consumers would be willing to pay additional costs. It is important to note that a significant proportion of the low-income population of Nairobi depends on informal supply chains. Careful consideration needs to be taken when making changes in these chains, as, if negative, they may reduce access to milk to vulnerable households and increase their risk of malnutrition. The results from this study therefore provide a practical framework that can be used to analyse the weaknesses and opportunities of the chains and overall dairy system, and to generate suitable policy interventions. Upgrade recommendations based on the finding of this study are shown in Table 2. Policymakers, when planning any development or mitigation measures in the value chains, would need to critically evaluate impact in each of the system segments or chain profiles. The importance of this has been underscored in other studies (Alarcon et al., 2017; FAO, 2011b) and has also been increasingly utilized to inform critical policy changes (Leksmono et al., 2006).

Our focus on the people and products revealed the complexity of interaction between people and products flows. The chains were principally made up of discrete individual entities (producers, retailers, traders) who were mainly operating on a small scale. This probably follows the country's dependence on smallholders that is seen along the entire value chain (KDB, 2014). For example, the largest proportion of suppliers for large processing companies was comprised of smallholders who had come together to form milk collection centres, dairy cooperatives or self-help groups and were not purely individual large-scale producers. This indicates the important role played by the small-scale producers in the overall national supply and their contribution to the complexity of the chains. Therefore, stakeholders should be cognizant of this while formulating any chain development and upgrade strategies.

The vast inter-linkages between the formal and informal chains observed in this study indicated that there were no obvious boundaries for operations, but that all chains were driven by consumer demand. For example, milk from some informal chains would flow into the formal chains when demand was higher. Viewed from a food safety perspective, this study suggests that food safety concerns in the informal chains would “spill-over” to the formal chains as suggested by other studies (Leksmono et al., 2006; Roesel and Grace, 2014). We therefore argue on the importance of enhancing health education to both systems. However, this cannot be effectively achieved without understanding the entry points into the systems.

Our findings on the production systems in the urban and peri-urban areas show important inefficiencies of the system and thus the overall inability to adequately supply the city's population. Production in both systems exhibited limited access to quality animal health services, ineffective breeding services, and inadequate feeding requirements as well as constrained housing owing to limited land. Although urban farming has gained prominence in the recent past, seen as a pathway to food security to the rapidly growing populations in cities (Lee-Smith, 2010), the underlying inefficiencies prevent the realization of such intentions. The population of Nairobi is projected to double by 2050 (Aubry et al., 2010); while demand for milk may triple by the same time (Herrero et al., 2014). This means that the city will be expected to produce >30 times the current production, which stands at 37 million litres per year (unpublished data, 2012). Hence, the deliberate enhancement of the supply chains emanating from outside the city will be critical. While there is need to enhance milk production to meet the current and the anticipated demand for dairy products, it is evident that opportunities for expanding production particularly in the city and peri-urban areas are limited owing to the prevailing system inefficiencies (Musa and Achola, 2015; Southall, 2005). Additionally, small-scale production in the city is more of a subsistence driven rather than business oriented activity. Therefore, the producers are not able to attract supportive services (extension, veterinary, breeding and business development) effectively. Consequently, productivity remains low. It has been shown elsewhere that organized groups are more likely to benefit from such services due to their enhanced social capital (Acharya et al., 2010); this was also observed in the current study where producers affiliated to dairy cooperatives accessed such services, as well as farm and domestic inputs on credit.

With the widening margin between milk production and demand in the city, alternative supply sources are inevitable. The readily sought alternative by the traders was the peri-urban and other distant farms from the rural areas. Currently, these alternative sources (peri-urban and rural areas) may appear to have enough milk to supply into the city. However, with the anticipated urbanization in rural Africa (United Nations, 2007) and the noticeable growth of major cities in Kenya following devolution of development through County system of governance (Kenya Government, 2010), the current supply may not be adequate to satisfy the anticipated demand. Instead, the peri-urban and the rural areas will concentrate on supplying the cities that would be coming up in their areas. This will further be confounded by the seasonality pattern for milk production in the country which is principally smallholder relying on rain-fed pastures (KDB, 2014). The system may have to rely in an increase of larger and more complex chains coming from outside the city. Understanding the current structure and functionality of the dairy system is therefore critical for policymakers in charge of planning and regulating the sector. The current structure of the dairy value chain will have to change to meet the anticipated rising demand. The policy interventions will need to address not only strategies for increasing production (more farms), but also that the system must become more resilient to externalities and efficient now and in the long run to particularly enhance milk production per cow, improve cold chains, promote value addition activities and support marketing channels to prevent losses.

Additionally, the structure for sourcing and transportation of milk by traders, which was reported to sometimes occur from over 200 km from the city, may not guarantee freshness and hygiene due to lack of cold chain and use of appropriate milk containers. It has also been observed that a new trend of supply into the African cities may arise from the regions perceived to have efficient supply chains and higher hygienic standards. The European Union (EU) for example, abolished the quota system of milk production in 2015, encouraging maximised milk output that may end up in surplus; which may find its way into these cities hence further complicating survival of the local dairy enterprises, particularly of small-scale stakeholders. The recent acquisition of 40% shares from Kenya's leading dairy processor by a major European firm is an early indicator of such penetration of the international dairy enterprises into the African dairy markets (Food Business Africa, 2015).

There have been attempts to organize the informal milk trading through formation of DTA by the government. Although this was meant to streamline milk marketing in the informal systems, this study established that traders affiliated to DTA have continued to operate at individual capacities and with similar practices to the non-DTA traders. It appears doubtful that the major objective was achieved. It seems that traders have no perceived benefits for joining the DTA as seen from the low membership and their unwillingness to participate in the business development training organized by the association. It is possible that even those traders who had joined DTA may have utilized this platform to legitimize their businesses rather than embracing the principle focus. The commodity group trading models, such as associations or cooperatives, are intended to facilitate product assembly, lobby for prices, and seek markets among others. These have particularly been successful in the rural/high milk production areas (Rademaker et al., 2016). However, promotion of dairy associations/ dairy cooperatives in the areas such as Nairobi city where there is ready market and the consumers are willing to pay for the milk may be difficult to thrive as evidenced by the low membership in the DTA. Additionally, platforms like dairy cooperatives become unpopular due to their inability to pay better prices and on timely manner as seen in the informal system which pays higher premiums on cash at delivery (FAO, 2011a; TechnoServe, 2008). The attempt to formalize the traders in order to enhance food safety (Leksmono et al., 2006), appears not to have achieved the desired objective because the dynamics between the formalized traders (DTA) and the non-DTA appeared to be similar.

Some of the limitations that would be important in the interpretation of results from this study were that data were mainly qualitative, gathered through narrations, and quantitative data through proportional estimation from the FGDs and key informant interviews. To address this limitation, we interviewed a wide variety of people representing the various segments of the value chain while ensuring adequate triangulation to minimize errors. The types of questions asked during the interviews also allowed for free discussions without leading participants to any specific answers. It was key that during the FGDs consensus was reached, following rigorous logical discussions and the results were also presented to other key informants in the system to assess for errors and validate the results. Finally, the scope of this paper was limited to mapping value chain structure, rather than all the other components of a value chain analysis; further elements of the value chain analysis are planned as part of further ongoing analyses.

## 5 Conclusion

This study provides an understanding of the structure of the dairy system operating in Nairobi. It has demonstrated the interdependency of the stakeholders involved in the inter-linked dairy chain profiles. As such, the study has demonstrated the need for holistic approaches and well - defined policy interventions that target every segment of the value chain in order to enhance the system's efficiency. The established structure, therefore, represents an essential framework for any interventions and it allows for future research to be conducted on system governance, upgrading, equity and food security and safety issues. This methodology presented here, based also on (Alarcon et al., 2017), further demonstrate a practical approach to map dairy systems.

## Ethical approval

Ethical approval for this study was obtained from the ILRI Institutional Research Ethics Committee (project reference: ILRI-IREC2014–04/1). ILRI IREC is accredited by the National Commission for Science, Technology and Innovation (NACOSTI) in Kenya. Ethical approval was also obtained from the Royal Veterinary College ethics committee (project reference: URN 2013 0084H).

## Conflict of interest

None.

## Figures and Tables

**Table 1:** People interviewed in the dairy value chain supplying Nairobi.

Chain node/Functions	People working in the chains	Activity	No. of FGDs (No. of people)	No. of KIs (No. of people)
Input supply	Feed manufacturers	Feed production, feed distribution, advisory on dairy cow feeding	–	1 (3)
Production	Dairy cow farmers in urban informal settlements (Kibera)	Milk production, selling of milk	1 (10)	–
	Dairy cow farmers in peri-urban areas (Dagoretti and Kikuyu)	Milk production, selling of milk	2 (14)	–
	Small scale Dairy Farmers Association	Offer advice to producers (production, breeding), linking producers to markets	–	1 (3)
	Kenya Livestock Producers Association	Create learning opportunities for producers through exhibitions, linking producers to financiers and markets	–	1 (2)
	Dairy Cooperatives (medium and large scale)	Milk assembly, bulking, cooling and transport of raw milk, extension services and inputs, offer credit facilities, and soft loans to producers	1 (7)	1 (1)
Milk collection and selling	Traders affiliated to the Dairy Trader Association	Extension and inputs, milk assembly, bulking and transport of raw milk, lobbying for policies	–	3 (3)
	Traders not affiliated to the Dairy Trader Association	Milk assembly, bulking and transport of raw milk	3 (19)	–
		Milk assembly, bulking, cooling and transport of raw milk, extension services and inputs, offer credit facilities, and soft loans to producers	1 (7)	1 (1)
Processing	Two largest milk processing companies based in Nairobi (Interviews done with managers of the companies)	Extension services, milk assembly, bulking, cooling and transport of raw milk, milk processing, value addition and distribution of processed milk products	–	6 (8)
Retailing	Supermarkets, traders, milk bars, restaurants, automated milk machines (ATM), roadside vendors, shops and kiosks	Processed and raw milk outlets, direct milk sales to consumers	2 (13)	2 (2)
People supporting and influencing the chains	Kenya Dairy Board	Set standards, inspect and license dairy enterprises, regulate dairy industry and facilitate trade	2(8)	2 (4)
	Directorate of Veterinary Services (head office)	Disease control, extension services to producers, facilitate trade	–	2 (2)
	Directorate of Livestock Production (head office)	Extension services to producers	–	1 (2)
Service providers/ Influencers	Livestock production officers (at sub-county level)	Provision of extension services (e.g. advice on animal management, housing, etc.)	7 (21)	–
	City council of Nairobi	License businesses	1 (6)	–
	Non-Governmental Organizations and Donor partners (Technoserve, USAID)	Extension services, strengthening producer groups / cooperatives	–	2 (4)
Total			21 (105)	23 (35)

**Table 2:** Potential upgrade recommendations for the Nairobi diary value chain.

Key message	Potential upgrade recommendations
• Large informal sector in operation, with numerous interlinkages with the formal sector.	Policies that allow regulating the informal sector without disturbing access of milk to low income households. Informal sector supplies a significant amount of milk through mobile retailers. Further research on the importance of these to distribute milk to low income consumers and their potential upgrade need to be considered.
• Low herd size of the majority of dairy farms in urban and peri-urban Nairobi, including in farmer in cooperatives. Plus 89% of farmers classified as long-term supplier to the large processing companies are also small scale. • Most farms operate independently (no association or outside of cooperatives) • Source replacement animals through neighbours and through rumours • Lack of used of veterinarians	These are likely to be less efficient (in terms of production and conversion of inputs, and their costs) that modern medium or large farms. Large processing companies could see their supply system more reliable if they develop programs to help their farmers increase their herd size and upgrade their farms. System upgrade is recommended to increase herd size, either through association of farmers, who could share cost of inputs, improve breeds, access to qualified veterinarians or animal health providers and the access to milk distribution chains. There is need for upgrading programs to create more cooperatives or motivate farmers to join in cooperatives. However, farmers using these as subsistence (so for own consumption) might not be able to do this.
• Lack of cold chain in milk transport. Milk rejected and given to pig farmers or sold a low price, indicating losses in revenue and system efficiency. • Use of plastic containers by many transporters in the informal sector • Use of public transport	Upgrade to improve cold chain and improve creation of milk chain from more distant areas where production could be more efficient Upgrade the type of containers. There is a need to understand the drivers for using the different type of equipment. Provide capacity to traders and farmers to efficiently transport their milk Need to understand to what extend the wastage of milk is a problem. Future research on this is recommended.
• Only 20% of traders are DTA • Only 40% of DTA have training	Require investigation of drivers on why traders are not joining the DTA. Upgrade is needed regarding how to include Non-DTA into the regulated system. Increase provision of training to also DTA and non-DTA is needed to ensure food safety practices and efficient operations of the chains.
• Lack of value addition by independent farmers, non-DTA traders and medium size cooperatives.	Training on this could help them to access better markets and prolong the life of their products. Careful consideration should be done, as this are the chains that most likely reach low-income consumers.
• Lack of chilling facilities for independent farmers and medium size cooperatives.	Policies to support loans or development of chillers could be considered.

## Annex 2

Housing, feeding, breeding and animal health services for the dairy cows kept in the informal and peri-urban areas of Nairobi

### Housing, feeding and watering:

Both the urban informal and peri-urban farmers described their farming systems as zero grazing. The housing facilities were mainly constructed adjacent to the farmers' main residential house because of insecurities associated with theft of livestock but also due to land scarcity. In some instances particularly in the urban informal, the cowsheds were said (and observed by researchers) to share the same roof with the owners, and only partitioned by a wall.

Livestock were described to be mainly stall fed on napier grass (*Pennisetum purpureum*), garden leftovers after harvesting, local grass (*Pennisetum clandestinum*), hay and kitchen leftovers. FGD with dairy cow farmers in the peri-urban areas identified own grown fodder and garden leftovers (maize stocks and banana stems) as the most common livestock feed in the peri-urban while the urban informal mainly utilized leftovers from markets and garbage from dumping sites (FGD dairy cow farmers in urban informal). Market leftovers were particularly fetched from the roadside vendors who routinely sell roasted maize, vegetables, fruits and green groceries. A small fee was said to be paid to the roadside vendors and the cartels of street children usually operating the dumpsites to facilitate the collection of these leftovers.

During dry seasons with fodder scarcity, some livestock owners purchased hay or cut pastures available by the roadsides. In addition, urban informal farmers reported to cut grass along the leaking sewer lines. Due to its high cost, farmers reported that hay was rarely fed alone, but usually mixed with any other available feeds. Almost all farmers reported to use commercial dairy meal, maize germ or bran during milking. Sweepings from poultry houses were also reported to be mixed with commercial feeds supposedly to increase milk production (FGD with farmers peri-urban).

Water provision for the dairy stock within urban informal settlements was mainly from piped water supplied by the city council, although sometimes wastewater derived from washing clothes or utensils was reported to be used. On the other hand, FGD with farmers in the peri-urban areas reported use of water mainly from boreholes within the neighbourhoods and sometimes from piped water, shallow wells and stored rainwater.

### Breeding services:

Artificial insemination (AI) and natural breeding methods were reported to be utilized by both farming systems, but with the former more commonly used than the latter. Although farmers reported their preference for AI due to perceived benefits of increased milk yields, they described natural breeding method as the most successful. They considered AI as a trial and error method whose success would probably occur after three or more trials or no success at all. Farmers in the urban informal settlements reported that the AI providers would sometimes inseminate three days after the cows had shown heat while in other instances they would inseminate a cow twice (morning and evening) apparently to increase the chances of conception. It was common practice in both farming systems for farmers to opt for natural breeding following such frustrations from AI. Some of the farmers within the FGD with urban informal reported that they did not attempt AI at all due to previous frustrations or from the experiences they see from the neighbours. For the peri-urban farmers affiliated to dairy cooperatives, AI services were reported to be pre-organized by the dairy cooperative so that farmers did not have to pay cash upon service. According to interviews with dairy cooperatives, large processing milk companies and with farmers in the peri-urban, recovery of such AI related costs were obtained from the monies accrued on the milk deliveries at the end of the month. However, some of the farmers from peri-urban still had experienced low conception rates of their cows attributing this to perceived low quality semen. Some of the semen used in the peri-urban was reported to be sourced from United States of America (USA) and Netherlands which was perceived to be of higher quality but majority of the farmers used locally produced semen from the Kenya Animal Genetics Resource Centre (KAGRC). Although reportedly expensive, few farmers in the peri-urban reported to use sexed semen from KAGRC and from Worldwide Sires (Non-governmental Organization) to maximize the chances of getting female calves.

### Animal health services:

FGD with farmers in the urban informal described that treatment of their dairy stock was mainly decided and implemented by the farmer, family member or neighbours who were perceived to have animal disease knowledge and experience in their treatment, and without consultation with animal health professionals. Farmers reported that drugs were purchased from Agrovets (retailing shops for agricultural chemicals and veterinary medicines) or used herbal medicines obtained from the neighbourhood. Some medium and large-scale farmers occasionally utilized para-veterinarians (those with certificate or diploma training on animal health) in treatment of their livestock according to LPOs, while others it was the farmers who selected their own treatment (as with small farms). Farmers explained that veterinarians (those with Bachelors' Degree in Veterinary Medicine) and other livestock extension officers (government staff) were never contacted, principally because they were unknown to farmers. In addition, farmers explained that they were not willing to seek government veterinary services because rumours in the neighbourhood indicated that keeping livestock in the city was illegal. Farmers therefore perceived that discovery of their enterprises by government officials would result to their arrest and confiscation of their livestock.

On the other hand, farmers in the peri-urban areas reported to obtain animal health services principally from para-veterinarians, but with some degree of services obtained from veterinarians and own treatment following purchase of drugs from Agrovets. Farmers in the peri-urban also expressed their misery in finding veterinarians whom they termed as very difficult to find and were mainly consulted as the last resort, primarily to solve difficult problems such as repeated treatment failures, disease outbreak situations or to determine the cause of death when caused by unknown aetiologies. Farmers affiliated to the dairy cooperatives reported that services were usually obtained from animal health care specialists contracted by the cooperatives although some of these farmers sought for the services privately at their own cost to avoid monthly deduction in their revenues.

For both production systems, the main information source on animal health were reported to be the Agrovet shops, people in annual livestock agricultural shows, the local radio, television and also other farmers, mainly through word of mouth.

## Annex 3

Potential upgrade recommendations for the Nairobi diary value chain
